# Structure-Based Discovery and Biological Assays of a Novel PRMT5 Inhibitor for Non-Small Cell Lung Cancer

**DOI:** 10.3390/molecules27217436

**Published:** 2022-11-01

**Authors:** Yingqing Chen, Mingyu Zhang, Anxin Wu, Xiaojun Yao, Qianqian Wang

**Affiliations:** 1 Chronic Disease Research Center, Medical College, Dalian University, Dalian 116622, China; 2 Dr Neher’s Biophysics Laboratory for Innovative Drug Discovery, State Key Laboratory of Quality Research in Chinese Medicine, Macau University of Science and Technology, Taipa, Macau SAR 999078, China

**Keywords:** PRMT5, non-small cell lung cancer, 3039-0164, structure-based virtual screening, molecular docking

## Abstract

Protein arginine methyltransferase 5 (PRMT5) is a popular anticancer target that regulates histone or nonhistone methylation and is linked to the development and poor prognosis of non-small cell lung cancer. PRMT5 inhibitors have shown great promise in clinical trials as a cancer therapy. However, most inhibitors reported recently act in a SAM-competitive mode and lack structural diversity. In this paper, a novel non-SAM inhibitor, 3039-0164, was discovered by the structure-based virtual screening method. The binding mechanism of 3039-0164 to PRMT5 was revealed via molecular docking and molecular dynamics simulations. 3039-0164 inhibited PRMT5 enzymatic activity, downregulated the expression of PRMT5 downstream target genes (FGFR3 and eIF4E), and blocked the activation of the PI3K/AKT/mTOR and ERK signaling pathways. The discovery of 3039-0164 provides precise and creative hit compounds for the design optimization of PRMT5 lead compounds in non-small cell lung cancer.

## 1. Introduction

Non-small cell lung cancer (NSCLC) is the most common and lethal primary lung cancer, which is difficult to detect and requires systemic treatment [1]. Despite recent advances in surgery, radiotherapy, and targeted molecular therapies, the prognosis of NSCLC remains very poor. There is an urgent need to identify new therapeutic targets to alleviate the disease symptoms.

Epigenetics is closely linked to cancer development, affecting gene expression and cellular function by regulating DNA or histone methylation, acetylation, phosphorylation, ubiquitination modifications, and chromatin remodeling [2]. It is significant to note that epigenetic dysregulation is a characteristic of various human malignancies [3]. Recently, researchers have paid a lot of attention to the novel idea of using epigenetic treatments to generate focused anticancer medications. In eukaryotes, protein post-translational modification (PTM) is a sort of chemical modification that enhances protein function by adding covalent chemical groups and is engaged in a range of epigenetic regulatory mechanisms [4]. One of the most common PTMs is arginine methylation, which occurs mainly on nuclear and cytoplasmic proteins, and this process requires the regulation of protein arginine methyltransferases (PRMTs) that target arginine residues [5,6]. Therefore, PRMTs have recently attracted a lot of attention. PRMTs are involved in several cellular life processes in cancers, including transcriptional activation, RNA splicing, DNA damage, and other responses [7]. Human-derived PRMTs have been classified into three types based on their catalytic activity: type I, II, and III. PRMT5 is a type II PRMT that catalyzes monomethylation or symmetric dimethylation of protein substrates on arginine residues [8] and participates in tumor cell proliferation, growth, development, and Golgi assembly [9,10,11]. PRMT5 is highly expressed in human lung cancer cells and tissues [11]. Sheng and Wang et al. [12] concluded that PRMT5 could promote lung cancer cell proliferation by regulating multiple signaling pathways. A growing body of evidence suggests that targeting PRMT5 has therapeutic value in the treatment of human lung cancer [11,13]. Even though various PRMT5 inhibitors have been reported, most of them are S-adenosyl methionine (SAM) competitive, which means that they are located at the SAM site [14,15,16]. The typical PRMT5 inhibitors representing different scaffolds and their pharmacological mechanisms are shown in Table 1. LLY-283 and AMI-1 are both SAM-competitive inhibitors. The former has the inhibitory effect by blocking MDM4 splicing regulation, which interacts with the PRMT5 residue Phe327 by its phenyl group, making it highly selective for PRMT5; the latter possesses a double anion structure that binds to the SAM site, whose interaction poly activity is established by its sulfonic acid group [14,17,18]. T1551 is a new non-SAM inhibitor. The benzene ring in its indole scaffold forms a cation–π interaction with SAM, which along with hydrogen bonding and π–π stacking forces explains the inhibitory activity of T1551 against PRMT5 [19]. JNJ64619178 as a dual SAM/substrate competition inhibitor binds to the PRMT5/MEP50 complex’s SAM and substrate pockets [20]. One of the proteolysis targeting chimera (PROTAC) degraders, MS4322, competes with the PRMT5 substrate and breaks down PRMT5 through PROTAC protein hydrolysis, inhibiting PRMT5 production [10]. EPZ015666 has an IC_50_ value of 22 nM, and its THIQ moiety forms the cation–π interaction with the positively charged methyl group in SAM, which makes a fundamental linkage, reduces SmD3 methylation and cell death, and shows a dose-dependent anticancer effect [21,22]. Additionally, a growing number of clinical investigations have proven that EPZ015666 can exhibit antitumor properties by inactivating PRMT5 in acute myeloid leukemia [23], lung cancer [19,22], and retinoblastoma [24,25], and is a novel and potential therapeutic target independent of the SAM binding site. These findings have greatly stimulated our interest in exploring novel non-SAM PRMT5 inhibitors.

In this paper, we used the virtual screening method based on the receptor structure to choose 158 candidates acting at the PRMT5 substrate binding site (at the location of EPZ015666). Through molecular docking and molecular dynamics simulations, it could be found that the identified 3039-0164 bound to PRMT5 in a stable manner mainly by hydrogen bonding and π–π interactions. The MTT experiment showed that 3039-0164 could inhibit cell viability of the A549 non-small cell lung cancer cell line. Furthermore, protein function assays showed that 3039-0164 downregulated the expression of two PRMT5 target genes, FGFR3 and eIF4E, and impeded the activation of the PI3K/AKT/mTOR and ERK signaling pathways. The discovery of 3039-0164 opens up new avenues for research and design optimization of PRMT5 inhibitors, which can help improve the prognosis of non-small cell lung cancer and other cancers in the clinic.

## 2. Materials and Methods

### 2.1. Virtual Screening Based on Protein Structure

The Schrödinger Maestro package (Schrödinger, LLC, New York, NY, USA; Schrödinger, 2015) [26] was used for the virtual screening. The protein receptor PRMT5 was derived from the Protein Data Bank (PDB ID: 4X61, https://www.rcsb.org/, accessed: 25 December 2021). The complex was first processed in the Protein Preparation Wizard module. All water molecules were removed, and the protonation state of all charged residues was adjusted to neutral pH. Then the docking grid was generated with EPZ015666 as the center. Since SAM has a cation–π interaction with EPZ015666 and affects PRMT5 inhibitor binding [21], the substrates are divided into two groups with/without SAM for enrichment factors’ (EFs) control assessment for subsequent screening. The sixteen EPZ015666 active derivatives selected in the previous study [27] were generated by DUD-E [28] in a 1:50 ratio of 800 decoys so that the relevant active factor and decoy molecules were docked near the EPZ015666 binding site. The electric field intensity was then calculated for the two groups of models 1 and 10% with/without SAM, respectively.

To eliminate the compounds with false-positive chemical and non-drug-like functional groups, before the virtual screening, the 1,671,908 compounds from the ChemDiv, Specs, and TargetMol databases were preliminarily screened based on the three pan-assay interference structures (PAINS) and one rapid elimination of swill (REOS) rules in Schrödinger Suite’s Canvas software. Meanwhile, the compounds meeting Lipinski’s rule of five were retained. The obtained compounds were then used for the virtual screening based on molecular docking. The top 10% (1706) of small molecules were clustered based on the Glide docking score, and the group size was set to 200. Finally, 158 compounds were selected for subsequent experimental examination by observing the binding pose of each ligand. All compounds were purchased from TopScience (Shanghai, China).

### 2.2. Molecular Docking and MD Simulations

Molecular docking was performed using the Schrödinger software package (Schrödinger, LLC, New York, NY, USA; Schrödinger, 2015). PRMT5 was selected from the Protein Data Bank (PDB ID: 4X61, https://www.rcsb.org/, accessed: 25 December 2021) and prepared using the Protein Preparation Wizard module, for example, hydrogenation and refinement of the structure, with the OPLS3 force field. Then, with EPZ015666 as the docking center, a docking grid box was generated. Next, the 3039-0164 small molecule structure file was downloaded from pubchem.ncbi.nlm.nih.gov/ and prepared in the Schrödinger software. Finally, molecular docking analysis was performed by the Glide module.

The Amber20 [29] software was used for MD simulations. For the PRMT5-SAM-3039-0164 complex, the FF19SB [30] and GAFF [31] force fields were employed, respectively. Steepest-descent/conjugate-gradient minimizations were initially applied to the systems for every 2500 steps, and then the systems were gradually heated in NVT ensemble from 0 to 310 K in 100 ps with a 10 kcal/(mol Å^2^) restrain on protein backbone atoms. The restrain was gradually decreased within 0.9 ns from 10 to 0.01 kcal/(mol Å^2^). Finally, without applying any constraints, three 200 ns MD simulations were run at 310 K and 1 atm. The Cpptraj [32] module of Amber20 was used to calculate the root mean square deviations (RMSD) and the contact analysis.

### 2.3. Cell Culture and Cytotoxicity Test

The non-small cell lung cancer cell line A549 (purchased from ATCC) was selected and cultured in RPMI 1640 medium containing 10% FBS (Gibco Products, Big Cabin, OK, United States) and 1% penicillin–streptomycin solution in a CO_2_ incubator at 37 °C containing 5% CO_2_. A total of 158 compounds obtained from the virtual screening were dissolved in DMSO and placed in a −40 °C environment. To rapidly identify compounds with strong inhibitory activity, the A549 cell line was first treated with 20 μM of each compound for 72 h. For the MTT assay, cells were first inoculated in 96-well 3000 cells/well microplates and incubated overnight to allow cell adhesion, and DMSO (10.0 µM) and different concentrations (2.5, 5.0, and 10.0 µM) were compared with compounds, which were studied at 24, 48, and 72 h. Then, 10 µL of MTT (5 mg/mL) was added to each for 4 h incubation at 3 °C, followed by 100 µL of acid isopropanol (10% SDS and 0.01 mol/L hydrochloric acid). Finally, the absorbance of the samples at 570 nm was measured with a microplate reader (Tecan US, Inc., Morrisville, NC, USA). Cell viability was calculated relative to untreated controls, and data conclusions were based on at least three independent experiments.

### 2.4. In Vitro Enzymatic Assays

PRMT5 enzymatic assay was performed as previously done by Ji et al. [33] at ChemPartner Shanghai (998 Harley Road, Pudong New Area, Shanghai, 201203, China). 3039-0164 was diluted to 10 concentrations to obtain IC_50_ values. PRMT5 and SAM/SAH were derived from BPS Bioscience (Cat. No. 51045) and Sigma (Cat. No. a7007-100 mg, and No. A9384-25MG). 3039-0164 was prepared as 10 mM of backup material in DMSO and diluted in DMSO to the final concentration. PRMT5 and substrate were incubated at the indicated concentrations of 3039-0164 in 384-well plates for 60 min at room temperature. Then, the remaining substrate of PRMT5 was labeled by adding the receptor and ligand solutions. The labeling procedure was reacted at room temperature for 60 min, and then the endpoint values were read using EnSpire Alpha mode. In the in vitro enzymatic assay, 1% DMSO was used as the control.

### 2.5. Western Blotting

Proteins were extracted from A549 cells using RIPA lysis buffer containing protease and phosphatase inhibitors. After two washes with cold PBS, the cell lysate (12,000× *g*, 4 °C) was centrifuged for 5 min, and the supernatant fraction was collected. The protein concentration was determined using the Bio-Rad protein assay kit (Bio-Rad, Philadelphia, PA, USA). The extracted proteins were separated in 10% SDS-PAGE gels (50 μg) and transferred to nitrocellulose (NC) membranes at 300 mA for 1 h at 4 °C. The proteins were then incubated with a primary antibody (1:1000) and a fluorescence-coupled secondary antibody (1:10,000). Additionally, eIF4E antibodies were obtained from Santa Cruz Biotechnology (Dallas, TX, USA); antibodies against total/phospho-AKT, total/phospho-ERK, and total/phospho-mTOR were obtained from Cell Signaling Technology (Danvers, MA, USA).

### 2.6. Statistics

Analyzed data were expressed through mean ± standard deviation. All data were statistically analyzed using GraphPad Prism 5.0. One-way analysis of variance (ANOVA) was used for multiple comparisons. *p* < 0.05 was considered statistically significant.

## 3. Results

### 3.1. Screening of Candidate Compounds by Structure-Based Virtual Screening

**Figure 1:**
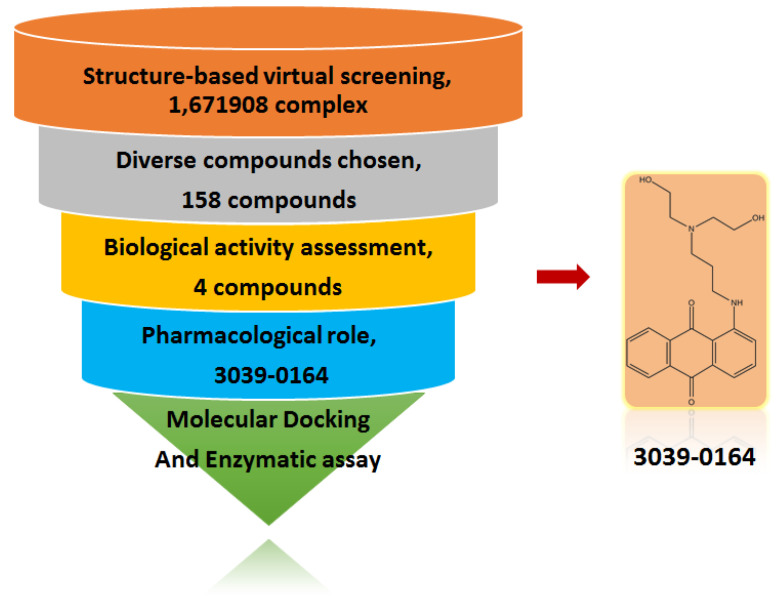
Schematic diagram of identifying 3039-064 by a structure-based virtual screening method.

**Figure 2:**
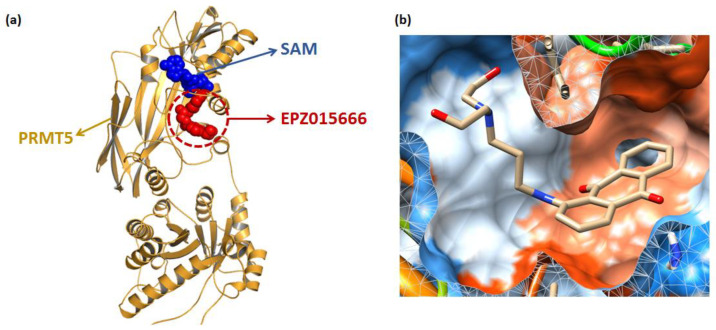
3039-0164 molecular docking. (**a**) Distribution of two co-crystalline compounds, SAM and EPZ015666, in PRMT5. The blue spherical part is SAM, and the red spherical model indicates EPZ015666. (**b**) The state of 3039-0164 is bound in the PRMT5 pocket. The blue region of the pocket is the hydrophilic part, and the orange region indicates hydrophobicity.

Structure-based virtual screening (SBVS) plays a key role in drug discovery research [34,35]. The method eliminates low-affinity candidate compounds by protein-ligand binding patterns and selects features of interest to further reduce the number of tested ligands for screening [36]. The SBVS workflow was indicated in Figure 1 in this paper. To identify the novel non-SAM PRMT5 inhibitors, the EPZ015666 pocket (Figure 2a) was chosen as the binding site. Since SAM has a partial interaction with EPZ015666 [16,37] and its effect is unknown, the enrichment factor was calculated after the docking preparation by setting the two complex modes with/without SAM. Additionally, the results showed that the 1% and 10% enrichment factors of the PRMT5-EPZ015666-SAM group were 44.6 and 8.7, respectively, which were higher than that of the PRMT5-EPZ015666 group (38.3 and 6.8). This shows that the presence of SAM increases the PRMT5’s affinity for the active compounds; hence, the PRMT5-EPZ015666-SAM complex was chosen for the screening.

Virtual screening was carried out for 1,671,908 chemical ligands with the previously established grid boxes. Following that, 1706 candidate compounds in the top 10% of the Glide score were submitted to 200-group cluster analysis. Compounds with more diversity, more interactions, and plausible binding patterns were chosen. Candidates with smaller molecular weights or lower ∆G_MMGBSA_ values were preferred, when structurally similar candidates were discovered. Based on these conditions, 158 small-molecule candidates were reserved for the further biological test. We performed MTT assays on these 158 candidate compounds. Results showed that in the A549 non-small cell lung cancer cell line, 3039-0164 showed a strong inhibitory effect. Meanwhile, its ∆G_MMGBSA_ value of 3039-0164 with PRMT5 was lower than the others (−76.2 kcal/mol). Therefore, 3039-0164 was selected for the following studies.

### 3.2. Molecular Mechanism of 3039-0164 Binding to PRMT5 

Molecular docking [38] is a mature analytical method focusing on the analysis of protein ligands, which predicts the binding mode and affinity based on the 3D protein structure, and has recently been used to study new inhibitors [39]. The binding mode of 3039-0164 with PRMT5-SAM is shown in Figure 2b, with a docking score of −8.51 kcal/mol. In Figure 2b, it is shown that 3039-0164 is a U-shape embedded in the protein pocket in a complementary shape, with its 9, 10-anthraquinone moiety occupying the hydrophobic end of the pocket and the two hydroxyl ends near the hydrophilic region.

**Figure 3:**
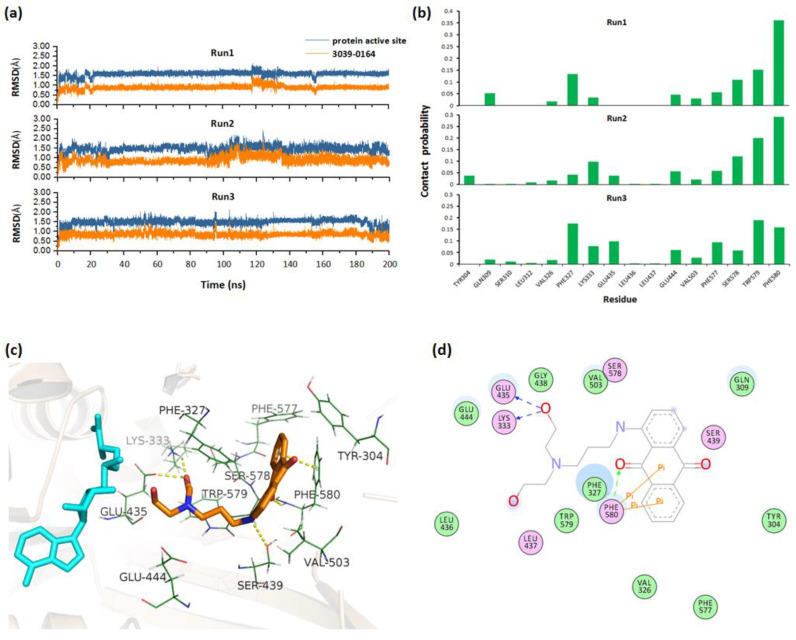
Molecular mechanism of 3039-0164 binding to PRMT5 based on MD simulations. (**a**) RMSDs for 3039-0164 and protein active residues along three parallel 200 ns MD simulations. (**b**) Contact probability of residues with 3039-0164 for three trajectories. (**c**) The 3D binding mode of 3039-0164 and PRMT5. Cyan and orange indicate the SAM substrate and 3039-0164 inhibitor, respectively. (**d**) The 2D binding mode of 3039-0164 and PRMT5.

To obtain the stable complex, three parallel 200 ns molecular dynamics simulations were performed for the docked PRMT5-SAM-3039-0164 complex. By monitoring the RMSD values of 3039-0164 and PRMT5 active site along the simulation, we could see that the system fluctuated stably after 140 ns (Figure 3a). Based on the stable trajectory, the atomic contact numbers of PRMT5 active site residues with the inhibitors were calculated. Figure 3b shows that the residues Phe580/327, Trp579/304, Ser578, Glu444, and Leu333 had relatively more contact with 3039-0164, indicating their importance for protein-inhibitor binding. Then, the stable PRMT5-SAM-3039-0164 structure was extracted to analyze the detailed binding mode. Figure 3c,d shows that 3039-0164 formed strong hydrogen bonds with the residues Lys333, Glu435, and Ser439. Additionally, the π–π stacking interactions were formed between the residue Phe580 and 3039-0164. These interacting factors work together to make the inhibitor bind to PRMT5 stably.

### 3.3. 3039-0164 Possesses Strong Cytotoxicity for A549 Cells

**Figure 4:**
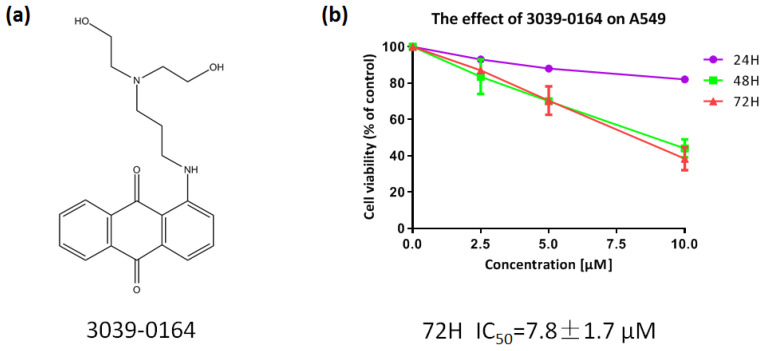
(**a**) Molecular structure of 3039-0164. (**b**) The cytotoxic effect of 3039-0164 on A549 cells and its IC_50_ value were detected by MTT assay.

PRMT5 was shown to be highly elevated in human lung cancer cells and tissues, and inhibiting its expression decreased the proliferation of tissue-cultured lung adenocarcinoma A549 cells significantly [40,41]. As a result, we chose the A549 non-small cell lung cancer cell line to investigate the inhibitory effect of 3039-0164 (Figure 4a). After preparing the compound into a range of concentrations (2.5, 5.0, 7.5, 10.0 µM), the survival rates of A549 cells were calculated after being treated for 24, 48, and 72 h (Figure 4b). With an IC_50_ value of 7.8 ± 1.7 μM and concentration dependence, 3039-0164 demonstrated the most significant antiproliferative effect on A549 cells in the 72 h continuous effect group. In summary, A549 was discovered to be extremely hazardous to 3039-0164.

### 3.4. 3039-0164 Inhibits PRMT5 Methyltransferase Activity and the Expression of Its Downstream Target Genes

**Figure 5:**
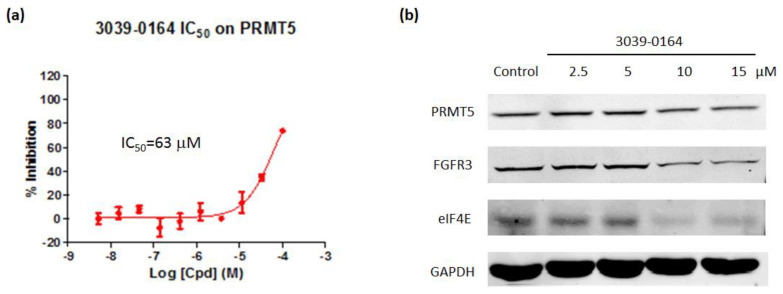
(**a**) 3039-0164 inhibition of PRMT5 methyltransferase activity and its IC_50_ value. (**b**) Protein expression levels of the oncogenes FGFR3 and eIF4E after treatment of A549 cells with different concentrations of 3039-0164 (2.5, 5.0, 10.0, and 15 µM). Western blot analysis was performed in 24 h with at least three independent experiments. Data are expressed as mean ± SEM (*n* ≥ 3), with * *p* < 0.05 for the control (DMSO-treated group) compared with the 3039-0164-treated group.

AlphaLISA assays were then used to investigate the inhibitory effect of 3039-0164 on PRMT5 activity. The results revealed that 3039-0164 inhibited this protein with an IC_50_ of 63 μM and a concentration-dependent impact (Figure 5a). This means that the PRMT5 methyltransferase activity was inhibited by this compound.

Many target genes are involved in PRMT5-induced lung small cell carcinoma. For example, knocking down the PRMT5 gene reduces the expression of FGFR3 and eIF4E [42]. Furthermore, FGFR3 and eIF4E have been found to be actively expressed in a variety of malignancies, including lung, prostate, and colorectal tumors, which cause disease [42,43,44]. The Western blot was performed to determine whether 3039-0164 inhibited the expression of FGFR3 and eIF4E target genes. The results are shown in Figure 5. The expression levels of FGFR3 and eIF4E proteins in A549 cells were significantly reduced by 3039-0164 at 10 and 15 μM doses. This indicates that 3039-0164 downregulates the expression of two downstream target genes, FGFR3 and eIF4E, by inhibiting PRMT5.

### 3.5. 3039-0164 Blocks the Activation of the FGFR3 Downstream Signaling Pathway

**Figure 6:**
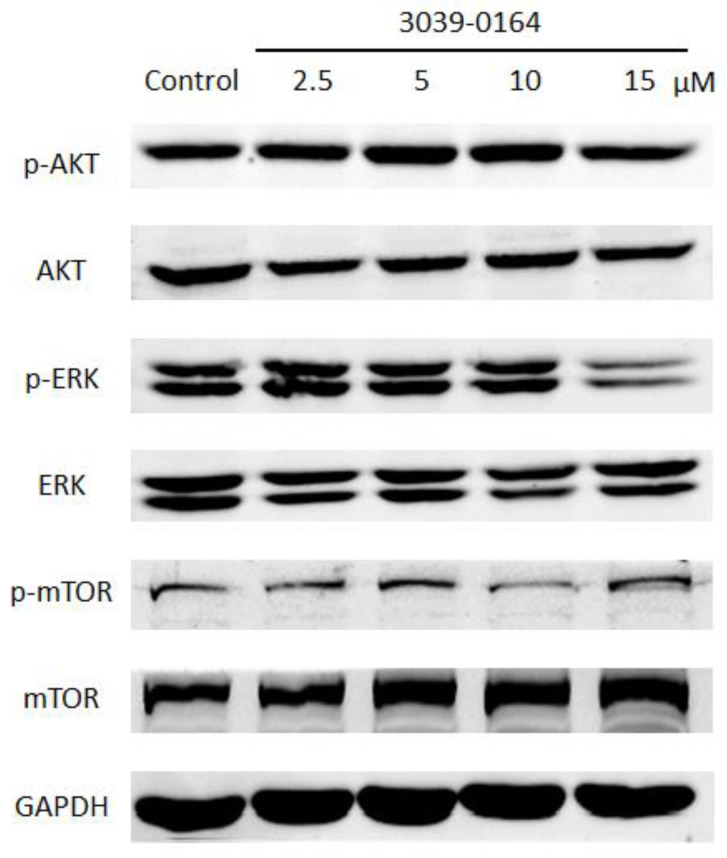
Protein expression levels of AKT, ERK, and mTOR among oncogene FGFR3 downstream signaling pathway factors after treatment of A549 cells with different concentrations of 3039-0164 (2.5, 5.0, 10.0, and 15 µM).

PRMT5 exhibits FGFR3-dependent regulation through the activation of AKT, ERK, and mTOR, and the activated FGFR signaling pathway plays an important role in promoting lung cancer cell proliferation [13,41,42]. We investigated whether the inhibitory effect of 3039-0164 on PRMT5 affected the activation of the AKT, ERK, and mTOR signaling pathways to learn more about the mechanism of cancer inhibition and the inhibitory effect of 3039-0164. When the concentration of 3039-0164 was 15 μM, phosphorylated AKT and ERK proteins were significantly suppressed, as shown in Figure 6. The expression of phosphorylated mTOR protein was considerably reduced at 10 μM. These findings suggest that 3039-0164 suppresses PRMT5 activity while suppressing the activation of the AKT, ERK, and mTOR signaling pathways downstream of PRMT5’s target gene FGFR3.

## 4. Discussion

Most advanced non-small cell lung cancer patients are prone to metastasis and have a poor prognosis, resulting in a high fatality rate [45]. Epigenetics regulates gene expression, leading to individual phenotypic variation, and its reversible regulatory nature makes it a popular candidate target when it comes to epigenetic therapy for various cancers [46,47]. In a previous study, our group identified the first BPTF bromodomain inhibitor, C620-0696, through protein expression abnormalities in the epigenetic dimension of lung cancer, and demonstrated its ability to inhibit the proliferation and migration of lung cancer cells [48]. PRMT5 is a promising predictive biomarker for lung cancer patients with high expression in lung tissues, according to numerous pieces of research published in recent years [49,50,51]. In this work, a novel non-SAM PRMT5 inhibitor, 3039-0164, was discovered, and it was shown that 3039-0164 inhibited PRMT5 methyltransferase activity and the activation of PRMT5 downstream target genes and pathways.

The PRMT5 has two binding pockets, one for the SAM methyl donor-binding site and the other for the EPZ015666 substrate-binding site. The majority of PRMT5 inhibitors discovered so far compete with SAM [16,37], and their inhibitory activity is not as potent as the original SAM molecule. Here, we concentrated our efforts on identifying novel PRMT5 inhibitors capable of binding to the EPZ015666 binding pocket. The potential inhibitor 3039-0164 was identified by a structure-based virtual screening approach. Following that, hydrogen bonds and π–π stacking interactions play a key role in the PRMT5-30390164 binding. Among them, PHE580, GLU444, SER578, and PHE327 are also the key active residues around EPZ015666 when binding to PRMT5 [19]. The binding conformation similarities between 3039-0164 and EPZ015666, as well as the mechanism of PRMT5 inhibition, were demonstrated to some extent by comparing the binding modes.

In in vitro studies, three features of 3039-0164’s anticancer effect against NSCLC were investigated: PRMT5 enzyme activity; expression of two target genes, FGFR3 and elF4E; and FGFR3-mediated downstream signaling pathways. The kinase experiment revealed that 3039-0164 inhibited PRMT5, but its IC_50_ value was high at 63 μM, which might be due to the fact that 3039-0164 was not directly targeting to inhibit this protein, and this process could entail the role of other components, which has yet to be investigated. Additionally, we found that the identified 3039-0164 could reduce the protein expression of the oncogenes FGFR3 and eIF4E by inhibiting PRMT5. Furthermore, when the inhibitor concentration was 10 μM, Western blotting results revealed a decrease in phosphorylated mTOR protein expression, whereas phosphorylated AKT and ERKs were simultaneously lowered at a 15 μM concentration of 3039-0164. This could be due to the PI3K signaling pathway’s signaling cascade amplification effect, which causes mTOR expression instability, but the combined phosphorylated AKT and ERK inhibition results could already indicate that 3039-0164 inhibited the activation of the PI3K/AKT/mTOR and ERK pathways in NSCLC cells. For the studied 3039-0164, it contains the anthraquinone substructure, which has been considered as a privileged scaffold in the drug discovery of various diseases, especially cancers, as reviewed in recent studies [52,53]. Some compounds with anthraquinone nucleus are also on the market, such as mitoxantrone, diacerein, and amrubicin [53]. Recently, with a series of biological evaluations, Volodina et al. [54] reported a thiophene-2-carboxamide derivative of anthraquinone (compound **8**) as a new potent antitumor chemotype, showing the promising anticancer effects of anthraquinone compounds. Despite this, the toxic assessment of this compound or the identification of toxic moieties, such as anthraquinone and the bis (2-hydroxyethyl) amino group, will be meaningful for drug safety. Simultaneously, in the next plan, further structural optimization is considered to improve its efficiency and selectivity for PRMT5. In the meanwhile, X-ray crystallography and biophysical techniques, such as microscale thermophoresis, isothermal titration calorimetry, or surface plasmon resonance, are expected to be used for verifying the direct targeted effects of promising inhibitors against PRMT5.

In conclusion, based on the virtual screening of a PRMT5-EPZ015666 substrate-binding pocket structure, this work finds a novel PRMT5 inhibitor, 3039-0164, with an anthraquinone moiety and demonstrates the influence of this inhibitor on cellular function through a series of biological tests. We hope that our research will spark some new ideas for improving the prognosis regimen of non-small cell lung cancer patients by optimizing the PRMT5 inhibitor design and developing drug-lead compounds.

## Figures and Tables

**Table 1:** Typical PRMT5 inhibitors representing different scaffolds and their pharmacological mechanisms.

Inhibitors	IC_50_	PDB id	Chemical Structures	Mechanisms of Action	Cancer Types	Ref.
LLY-283	20 nM	6CKC	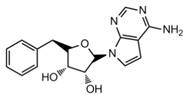	Occupied the SAM pocket; inhibition of MDM4 splicing regulation; phenyl occupancy of the Phe327 side chain, which may make it highly selective for PRMT5.	Glioblastoma	[14,18]
AMI-1	8 µM	-	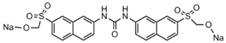	It has a double anion structure that binds to the SAM site; the interaction poly activity established by its sulfonic acid group makes it inhibit the test enzyme.	Rhabdomyosarcoma; lung cancer	[17]
T1551	34 µM	-	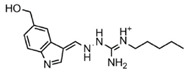	Through cation–π interactions with SAM, π–π interactions with Phe327, and hydrogen bonding with some residues.	Non-small cell lung cancer	[19]
EPZ015666	22 nM	4 × 61	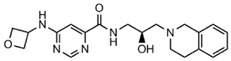	Substrate competitive; the THIQ group interacts with the cation–π formed by the partially positively charged methyl group of SAM.	Multiple myeloma; retinoblastoma; Mantle cell lymphoma	[22,24,25]
JNJ-64619178	0.1 nM	6RLQ	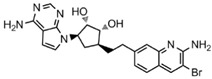	Occupies both SAM and substrate-binding sites; produces high affinity and, therefore, does not interact with MTAP-deficient cancer cell-specific complexes.	Acute myeloid leukemia; non-small cell lung cancer; pancreatic	[20]
MS4322	18 nM	-	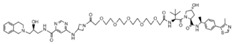	PROTAC degraders; competes with EPZ015666 for PRMT5 substrate binding sites, and reduces PRMT5 expression.	Non-small cell lung cancer; cervical cancer; glioblastoma	[10]

## Data Availability

The data presented in this study are available on request from the corresponding author.
